# Comparison of two methods of measuring the urinary protein concentration for the determination of the urinary protein to creatinine ratio in various animal species

**DOI:** 10.17221/30/2024-VETMED

**Published:** 2024-08-29

**Authors:** Simona Kovarikova, Jana Blahova, Ivana Vanova, Petr Marsalek

**Affiliations:** ^1^Department of Animal Welfare and Protection and Veterinary Public Health, Faculty of Veterinary Hygiene and Ecology, University of Veterinary Sciences, Brno, Czech Republic; ^2^Department of Physiology, Faculty of Veterinary Medicine, University of Veterinary Sciences, Brno, Czech Republic

**Keywords:** cat, dog, guinea pig, horse, proteinuria, UPC

## Abstract

Determination of the urinary protein-to-creatinine ratio (UPC) is an important tool in the quantification of proteinuria in animals. However, the result may be affected by the different methods of determining the urinary protein concentration. The aim of this study was to compare the turbidimetric method using benzethonium chloride and the colorimetric method using pyrogallol red in the measurement of the urinary protein concentration in dogs, cats, guinea pigs and horses. A total of 464, 192, 216 and 119 urine samples from dogs, cats, guinea pigs and horses were examined in the study, respectively. The group consisted of animals of both sexes and different ages, and, in the dogs and cats, it included both healthy animals and those with various health problems. In the group of horses and guinea pigs, only clinically healthy animals were included. A total of 347, 185, 103 and 100 samples from the dogs, cats, guinea pigs and horses were used in the statistical analysis; the other values were excluded as they were below the detection limit. According to the Passing-Bablok analysis, there was a significant constant and proportional difference in the horses. In the dogs, cats and guinea pigs, there was a significant constant difference, but no proportional difference. The Bland-Altman method showed significant bias between the two methods in the horses and cats, but not in the dogs and guinea pigs. In the dogs and cats, the agreement between the two methods was tested and expressed as Cohen’s kappa (κ). In the cats, it was almost perfect for the proteinuric samples (κ = 0.823 3) and significant for the non-proteinuric samples (κ = 0.804 9). In the dogs, the agreement was significant for the non-proteinuric samples (κ = 0.621 5) and only moderate for the proteinuric samples (κ = 0.527 5). The influence of the method used to determine the urinary protein concentration should be taken into account when evaluating the UPC. Repeated examinations in one patient should be performed with the same method.

The urinary protein-to-creatinine ratio (UPC) assessment is a traditional tool used for the quantification of proteinuria that reliably replaces the impractical 24-hour urine collection followed by protein concentration measurement (Monroe et al. 1989; Adams et al. 1992). It is commonly used in dogs and cats and is gradually becoming a way of describing the renal function in other animal species (Uberti et al. 2009; Harley and Langston 2012; Hokamp and Nabity 2016; Cernochova et al. 2020). Three different methods are commonly used to determine the protein concentration in urine: turbidimetric using benzethonium chloride and colorimetric based on the reaction with pyrogallol red or Coomassie brilliant blue. None of these methods is considered as a reference and all the methods are used in scientific publications (Giraldi et al. 2018; Moyle et al. 2018; Vientos-Plotts et al. 2018; Jillings et al. 2019; Mortier et al. 2023a). However, the results of recent studies show that different methods may give different results and thus influence the clinical decisions (Rossi et al. 2016; Giraldi et al. 2018; Mortier et al. 2023b). Thus, the aim of this study was to compare turbidimetric and colorimetric methods for the determination of the protein concentration in the urine of dogs, cats, guinea pigs and horses.

## MATERIAL AND METHODS

### Animals and collection of urine samples

This is a study looking at the analytical part of the sample analysis and therefore urine samples from animals were included in the study regardless of the breed, sex, age or health status.

For horses and guinea pigs, the urine samples were from healthy individuals and were obtained by normal voiding. In dogs and cats, the samples were obtained not only from healthy individuals, but also in private practice from patients with various health problems during routine healthcare. The urine samples from the canine and feline patients were obtained by normal voiding, catheterisation or cystocentesis. The samples were stored in Eppendorf-type tubes, frozen at –20 °C for a maximum of one month. After thawing, they were centrifuged and only the supernatant was used for the subsequent analysis.

### Evaluation of urine samples

The urinary protein and creatinine concentrations were determined using commercial kits and all the analyses were performed on an automated biochemical analyser Konelab 20i (ThermoFisher Scientific, Waltham, MA, USA). The urinary protein concentration was measured by both the turbidimetric and colorimetric methods. The principle of the turbidimetric method was the reaction of protein with benzethonium chloride with a limit of detection 68 mg/l (Urine/CSF Protein; Abbott GmBH, Wiesbaden, Germany). The colorimetric method consists of the reaction of protein with pyrogallol red and molybdate (total protein – urine, liquor; Biovendor, Brno, Czech Republic). The limit of detection of this method is 20 mg/l. The values of the urinary protein concentration below the limit of detection (LOD) were excluded from the statistical analysis. The urinary creatinine concentration was determined using the Jaffé method (Creatinine; Biovendor, Brno, Czech Republic). In the dogs, cats and horses, the urine sample for the assessment of the creatinine concentration was diluted prior to the analysis (50 μl of urine sample + 2 450 μl of ultrapure water). In the guinea pigs, a non-diluted urine sample was used. Certified reference materials of the total protein – urine/liquor and BioCal (Biovendor; Brno, Czech Republic) were used for the protein and creatinine calibration, respectively. The quality control was performed with each series of measurements using the certified reference materials TruLab Urine 1 and TruLab Urine 2 (Diasys, Holzheim, Germany).

The urine protein to creatinine ratio was then calculated for both methods and was designated as UPC-A (for the turbidimetric method) and UPC-B (for the colorimetric method). The first step was to convert the results to the same units (mg/dl). For the protein, the mg/l was divided by 10, for the creatinine, the mmol/l was multiplied by a factor of 11.3. Subsequently, the ratio was calculated. The International Renal Interest Society (IRIS) classification system for proteinuria was used in the dogs and cats. Samples with a UPC lower than 0.2 were classified as non-proteinuric. Borderline proteinuric samples had values between 0.2 and 0.5 in the dogs and had values between 0.2 and 0.4 in the cats, respectively. Samples with a UPC higher than 0.5 and 0.4 in the dogs and cats were classified as proteinuric.

### Statistical analysis

The protein concentration values that were below the limit of detection were excluded from statistical evaluation. The data were tested for normal distribution using the Kolmogorov-Smirnov test. Bland-Altman plot and Passing Bablok regression were used to evaluate whether there is a systematic bias between the UPC-A and UPC-B method. The statistical significance was accepted at *P* < 0.05. In the dogs and cats, the degree of agreement between the UPC-A and UPC-B method whether the results were non-proteinuric or proteinuric was assessed using Cohen’s κ coefficient, which corrects the observed agreement rate for the probability that the agreement is due to random chance. A coefficient κ value of 1.0 indicates perfect agreement between results, whereas a value of κ ≤ 0.0 indicates that any agreement is due to random chance alone. The strength of agreement using the coefficient was assessed by Cohen as follows: for κ = 0.81–1.0 values, agreement is considered almost perfect, for κ = 0.61–0.80 values, agreement is considered significant, for κ = 0.41–0.60 values, agreement is considered moderate, for κ = 0.21–0.40 values, agreement is considered fair, and for κ ≤ 0.20 values, agreement is considered none to slight (McHugh 2012). For visualisation of the IRIS classification changes between the methods, a Sankey diagram was used (Fluorish; Canva UK Operations Ltd, London, UK).

## RESULTS

**Figure 1:**
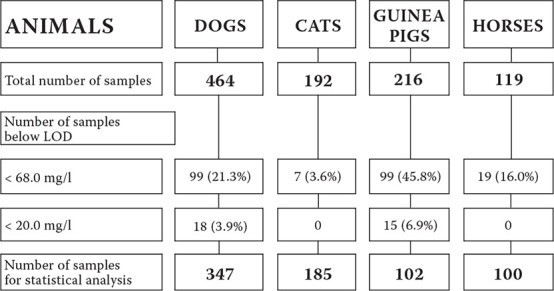
Numbers of samples examined by both urinary protein measurement methods, the numbers of samples excluded from the statistical analysis and the resulting number of samples evaluated in the dogs, cats, guinea pigs and horses Some samples were excluded from the study because the values were below the LOD of each method. Samples with values below 68 ml/l determined by the turbidimetric method had values both below and above 20 mg/l measured by the colorimetric method. In the case of the samples below the LOD in the colorimetric method (< 20 mg/l), these were above the LOD in the turbidimetric method (> 68 mg/l) LOD = limit of detection

A total of 464 urine samples from dogs, 192 urine samples from cats, 216 urine samples from guinea pigs and 119 urine samples from horses were examined in the study. A total of 117 samples (25.2%) from dogs, 7 samples (3.6%) from cats, 114 samples (52.8%) from guinea pigs and 19 samples (16.0%) from horses with values below the limit of detection were excluded from the statistical analysis (Figure 1). The descriptive characteristics of the groups are presented in Table 1.

**Table 1:** Concentrations of the urinary protein measured by the turbidimetric (Protein-A) and colorimetric (Protein-B) methods, urinary creatinine concentrations, and calculated urinary protein-to-creatinine ratio (UPC-A for the turbidimetric method and UPC-B for the colorimetric method, respectively) in the dogs, cats, horses and guinea pigs

Animals	Protein-A mean range (mg/l)	Protein-B mean range (mg/l)	Creatinine mean range (mmol/l)	UPC-A mean range	UPC-B mean range
Dogs (*n* = 347)	205.8 68.0–8 253.6	221.7 21.5–3 453.2	18.3 1.5–69.5	0.10 0.02–4.56	0.11 0.01–3.76!
Cats (*n* = 185)	350.4 69.1–1 847.7	369.2 39.7–6 003.7	22.2 1.1–56.4	0.17 0.02–2.98	0.18 0.04–7.37
Guinea pigs (*n* = 102)	188.5 73.3–6 184.2	197.1 72.0–6 875.9	3.1 0.3–53.9	0.60 0.08–17.52	0.67 0.07–75.21
Horses (*n* = 100)	175.8 74.3–1 884.5	175.3 21.4–1 659.7	19.7 2.6–57.3	0.10 0.03–3.55	0.08 0.03–3.13

UPC = urinary protein-to-creatinine ratio

**Figure 2:**
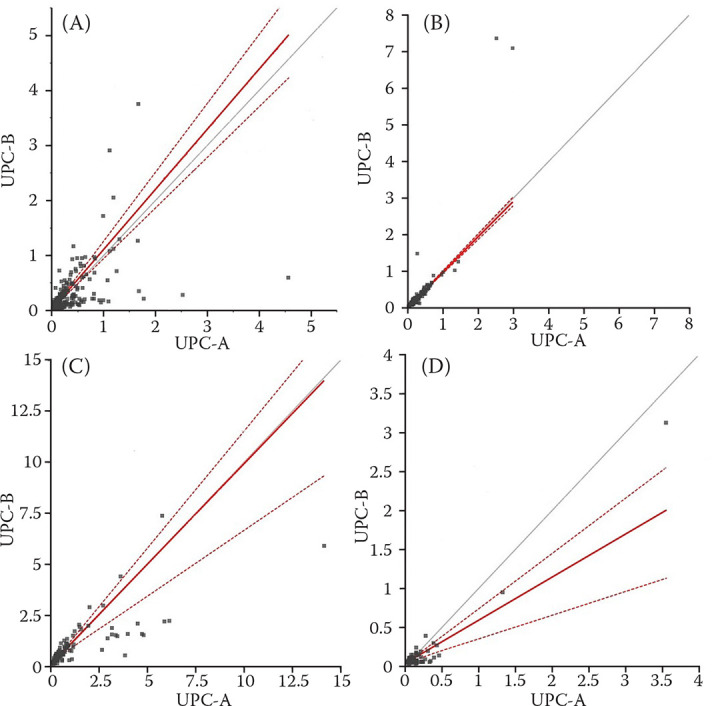
Comparison of the UPC-A (turbidimetric) and UPC-B (colorimetric) method using the Passing Bablok regression (A) Comparison for dogs (*n* = 347). (B) Comparison for cats (*n* = 185). (C) Comparison for guinea pigs (*n* = 102). (D) Comparison for horses (*n* = 100). The red line is the Passing Bablok fit, the grey line is the best fit and the red dotted lines represent the 95% confidence interval UPC = urinary protein-to-creatinine ratio

The Passing-Bablok regression method showed, for the determination of the UPC in horses, that the 95% confidence interval (CI) for the intercept and the 95% CI for the slope did not include the value zero and one, respectively, which means there was a significant constant and a significant proportional difference between the two methods. In the case of the determination of the UPC in the cats, dogs and guinea pigs (Figure 2), the 95% CI for the intercept of the Passing Bablok regression did not include the value zero while the 95% CI for the slope of the Passing-Bablok regression included the value one, which means there was a significant constant difference, but no significant proportional difference between the two methods.

**Figure 3:**
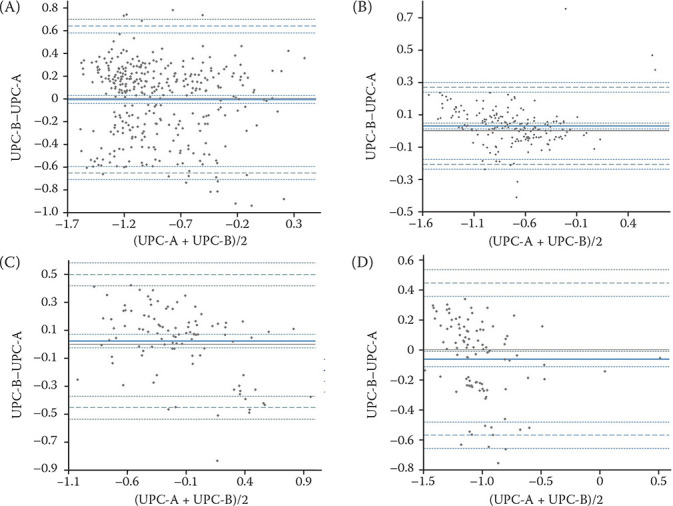
Comparison of the UPC-A (turbidimetric) and UPC-B (colorimetric) method using the Bland-Altman plot (A) Comparison for dogs (*n* = 347). (B) Comparison for cats (*n* = 185). (C) Comparison for guinea pigs (*n* = 102). (D) Comparison for horses (*n* = 100). The blue line is the mean of the UPC-B – UPC-A differences, the grey line is the line of equality, the blue long dotted lines represent the limits of agreement and the blue short dotted lines represent the 95% confidence interval UPC = urinary protein-to-creatinine ratio

Since the data did not meet the condition of normal distribution, they were log_10_-transformed for the analysis by the Bland-Altman method. The Bland-Altman method showed that the bias between the two methods for the determination of the UPC in the horses and cats is significant, because the line of equality is not within the 95% CI of the mean. The Bland-Altman method showed that the bias between the two methods for the determination of the UPC in the dogs and guinea pigs (Figure 3) is not significant, because the line of equality is within the 95% CI of the mean.

**Figure 4:**
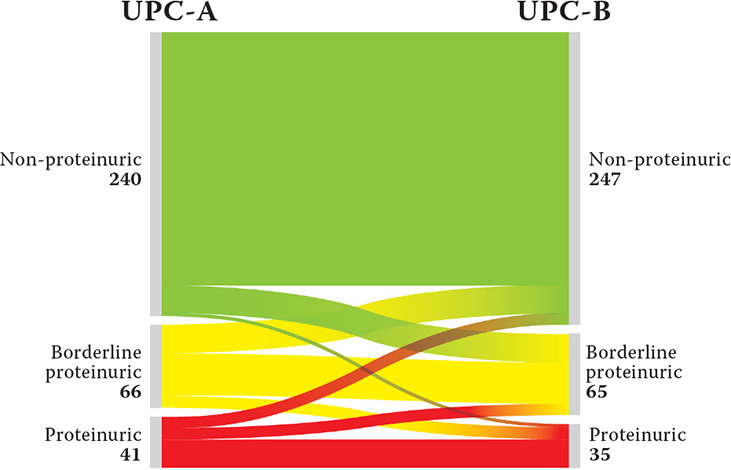
Classification of the samples according to the IRIS proteinuria substages based on the results obtained by the two methods (UPC-A – turbidimetric, UPC-B – colorimetric) and the expression of the change in the classification in the dogs IRIS = The International Renal Interest Society; UPC = urinary protein-to-creatinine ratio

According to the IRIS classification, using the turbidimetric method, 69.2%, 19.0%, and 11.8% of the canine samples were non-proteinuric, borderline proteinuric, and proteinuric, respectively; according to the colorimetric method, they were 71.2%, 18.7%, and 10.1%, respectively. However, 73 urine samples (21.1%) had a change in classification (Figure 4).

**Figure 5:**
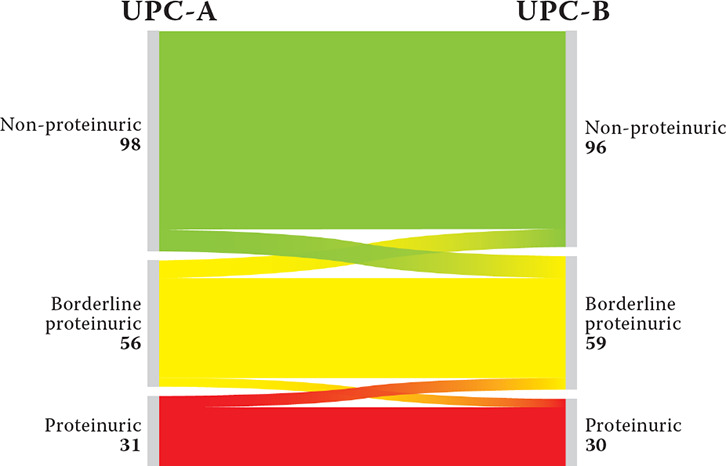
Classification of the samples according to the IRIS proteinuria substages based on the results obtained by the two methods (UPC-A – turbidimetric, UPC-B – colorimetric) and the expression of the change in the classification in the cats IRIS = The International Renal Interest Society; UPC = urinary protein-to-creatinine ratio

In the cats, based on the turbidimetric method, 60.0% of the samples were non-proteinuric, 30.3% of the samples were borderline proteinuric, and 16.7% of the samples were proteinuric; based on the colorimetric method, 51.9% of the samples were non-proteinuric, 31.9% of the samples were borderline proteinuric, and 16.2% of the samples were proteinuric. A change in classification occurred in 27 feline samples (14.6%) (Figure 5).

Testing the degree of agreement between the UPC-A and UPC-B method whether the results are non-proteinuric or proteinuric was assessed using Cohen’s κ coefficient. The proteinuric and borderline proteinuric results were combined to assess the degree of agreement on whether the samples were non-proteinuric. The non-proteinuric and borderline proteinuric results were combined to assess the degree of agreement on whether the samples were proteinuric. Almost perfect agreement (κ = 0.823 3) was found for the proteinuric samples of the cats and significant agreement (κ = 0.804 9) was found for the non-proteinuric samples of the cats. Moderate agreement was found for the proteinuric (κ = 0.527 5) and significant for the non-proteinuric (κ = 0.621 5) samples of the dogs.

## DISCUSSION

The differences between the methods of the urinary protein determination are very important from an analytical point of view. However, the current guidelines recommend specific cut-off values that do not take the possible differences between methods into account (Grauer 2011). To our knowledge, no study has yet been performed a comparing colorimetric determination with pyrogallol red (PRM) and a turbidimetric determination using benzethonium chloride (BTC) in different animal species. However, there are reports of differences between the two colorimetric methods [pyrogallol red, and Coomassie brilliant blue (CBB)] in dogs and cats, with the CBB method providing significantly higher urinary protein concentrations than the PRM method. Agreement in the staging samples according to the IRIS guidelines was good in the cats, and moderate in the dogs (Rossi et al. 2016; Giraldi et al. 2018). In a recent study, urine obtained from cats was sent to different commercial laboratories using either the colorimetric determination or turbidimetric determination and the UPC values obtained did not differ significantly (Mortier et al. 2023b). Nevertheless, the agreement on the IRIS substages between the laboratories was only moderate, when 253/360 (70%) of the UPC comparisons showed a classification within the same IRIS proteinuria substage.

Variations in the results obtained by different methods may be due to different reactions of the reagents to the proteins present in the urine. Colorimetric methods showed a constant underestimation of globulin when compared to albumin (Watanabe et al. 1986) and also with the frequent measurement of nonprotein nitrogenous waste (Marshall and Williams 2000). Falsely low protein results in very high protein concentrations have been reported for the benzethonium chloride method due to the formation of protein aggregates (Crofton 1989), thus Yilmaz et al. (2008) proposed the dilution of samples with a protein concentration > 2 500 mg/l when this method is used. In our dataset, we had only a few such high values measured by the benzethonium chloride method and only in the group of dogs. If we compare them with the PRM, they are higher, which is in contrast to the previous statement. Thus, further studies should be conducted on a larger group of urine samples with high protein concentrations. Differences in the use of PRM and BTC were noted in the examination of human urine samples and were influenced by the use or omission of sample centrifugation. The colorimetric PRM method was less affected by sample centrifugation than the BTC method (Surer et al. 2014). In our case, however, all the samples were treated the same (all were centrifuged), so this cannot be the reason for the differences. The differences in our results obtained by the two different methods are most likely due to the variable response to the protein present in the urine. In our case, the study included samples from different species of animals as well as from clinically healthy animals and patients with different health problems, so that a high variability in the urinary protein pattern can be expected. The differences between the methods are particularly evident in the samples with a high protein content, and, in this case, this may have important clinical implications.

In these cases, it would be useful to supplement the examination with urine protein electrophoresis, which could provide indications for further research into which proteins the methods are more responsive to.

Thus, laboratories should provide information not only on the method of determining the protein in the urine but also on the limitations of specific methods. In our case, the limit of detection played a major role. It was 68 mg/l for BTC and 20 mg/l for PRM. In all animal species, we had samples where the protein value was below the LOD, the largest number of such samples was in the guinea pigs and dogs. In the statistical analysis of the results, the values below the LOD can be handled in several ways. Options include deletion, simple substitution or distribution methods. However, simple substitution can only be used in cases where less than 15% of the data in the set is below the limit of detection. In our case, only samples originating from the cats would meet this, where only 7 samples (3.6%) were below the LOD. As our aim was not to determine specific ranges, but to compare two methods, we chose to delete such data below the LOD. It is important to know the limit of detection also from a clinical point of view. This is because, under certain circumstances (very low urinary creatinine concentration), even values below the LOD could result in significant proteinuria when substituted into the UPC formula. The BTC method with a higher limit of detection appears to be inappropriate for the assessment of proteinuria in guinea pigs because almost half of their samples were below the LOD and thus excluded from the study.

In the dogs and cats, we could also compare the results obtained by the two methods in terms of the IRIS classification of proteinuria. A total of 73 dogs (21.1%) and 27 cats (14.6%) had a change in proteinuria classification. While in the cats, the changes were within the adjacent categories, in the dogs, the changes were from the non-proteinuric to proteinuric individuals and *vice versa*. The different classification of proteinuria has clinical implications – it affects the decision-making process for the possible initiation or continuation of antiproteinuric therapy. In cats with chronic kidney disease, we know that even a UPC > 0.2 has a negative effect on the survival time compared to cats with a UPC < 0.2 (Syme et al. 2006). Therefore, such values should be interpreted with caution and in the case of unclear results, it is recommended to repeat the test to confirm the results or assess the progression, if necessary. Thus, our study shows that it is very important to perform this repeat examination in the same laboratory. This is also in line with the results of a previous study in which only 3% of samples changed classification when re-examined in the same laboratory (Mortier et al. 2023b). Our results, together with previous studies comparing different methods of urine protein determination, show that it would be preferable not to work with universal cut-off values, but to have a range for each used method of urine protein determination.

The limitation of this study is the inclusion of only healthy guinea pigs and horses. In the case of the dogs and cats, we see that there are greater differences between the proteinuric samples and this is lacking in the horses and guinea pigs and we are unable to assess this relationship better.

Our study showed that the results of the protein concentration in the urine obtained by colorimetric and turbidimetric methods differ. Differences also exist between species, which is probably due to the different responses to the proteins present in the urine. Unfortunately, we are unable to say which method is more accurate. Urine protein electrophoresis could clarify the behaviour of the methods and determine which of the methods under investigation is more suitable for the detection of the specific proteins in urine. Differences in the results obtained may also have a clinical impact, where the classification of proteinuria may be changed. Thus, one laboratory should be used for the assessment of the proteinuria when a single patient is being re-examined. The detection limit of a particular method is also important. According to this study, the turbidimetric method with a higher limit of detection is not suitable for guinea pigs and should be used with caution in dogs.
